# *Bai-Hu-Tang*, Ancient Chinese Medicine Formula, May Provide a New Complementary Treatment Option for Sepsis

**DOI:** 10.1155/2013/193084

**Published:** 2013-05-15

**Authors:** Chien-Jung Lin, Yi-Chang Su, Cheng-Hung Lee, Tsai-Chung Li, Yun-An Chen, Sunny Jui-Shan Lin

**Affiliations:** ^1^Department of Chinese Medicine, National Defense Medical Center, Tri-Service General Hospital, Taipei 11490, Taiwan; ^2^Graduate Institute of Chinese Medicine, College of Chinese Medicine, China Medical University, No. 91 Hsueh-Shih Road, Taichung 40402, Taiwan; ^3^School of Chinese Medicine, College of Chinese Medicine, China Medical University, Taichung 40402, Taiwan; ^4^Graduate Institute of Biostatistics, College of Public Health, China Medical University, Taichung 40402, Taiwan; ^5^Department of Healthcare Administration, College of Health Science, Asia University, Taichung 41354, Taiwan

## Abstract

*Bai-Hu-Tang* (BHT) has been broadly applied to treating the early stage of acute infection with systemic inflammation for two thousand years in Chinese medicine. We explore whether BHT is beneficial in treating sepsis and its effects on proinflammatory cytokine, interleukin-6, and anti-inflammatory cytokine interleukin-10, in which both play key roles in the progress of sepsis. Thirty-six male Sprague-Dawley rats were randomized into six groups, with cecal ligation and puncture (CLP) performed in all but the sham-control group. Rats in CLP + BHT-L_6_ and CLP + BHT-H_6_ groups, respectively, received a low (0.45 g/kg) and high doses (0.9 g/kg) of BHT, 6 hrs postoperatively. CLP + BHT-L_12_ and CLP + BHT-H_12_ groups, respectively, received low and high doses of BHT, 12 hrs postoperatively. Sham-control and sepsis-control groups received distilled water (1 mL) as vehicle, 6 hrs postoperatively. Serial blood samples were drawn before operation, as baseline, and at 4, 8, and 12 hrs postoperatively for IL-6 and IL-10 assay. All rats were monitored for 3 days for survival study. Rats in the CLP + BHT-H_6_ group had significantly higher survival rate (80%) and significantly lower levels of both IL-6 and IL-10 at 12 hrs postoperatively than those in the sepsis-control group. Results suggested that BHT may be a new complementary treatment option for sepsis.

## 1. Introduction

The term “translational research” first appeared in PubMed in 1993, and it has cropped up in hundreds of articles each year after 2000. Relatively recent, a chasm has opened up between biomedical researchers and the patients who need their discoveries [1, 2]. Enormous resources have been thrown into biomedical research, but the resulting commensurate gains are not as expected [1]. To bridge the gap between basic and clinical research, thriving treasures recorded in the Chinese medicine literature are worthy to be refined to provide promising targets since series of theoretical systems and practical experience of Chinese medicine were constructed through fighting diseases in the past thousands of years.

Sepsis is the leading cause of death in intensive care patients, with a reported incidence of more than 750,000 people each year in North America and Europe [3, 4]. Despite major advances in the understanding of its pathogenesis, no satisfactory therapy has emerged [5, 6]; mortality from severe sepsis remains high at approximately 28% [3].

The most prominent pathological feature of sepsis is the exuberant, poorly regulated cytokine-mediated inflammatory response to microbial products [7]. Release of proinflammatory cytokines prolongs the inflammatory reaction [8] and leads to organ injury or dysfunction [9–12]. To prevent severe damage, anti-inflammatory cytokines are released. With overproduction of anti-inflammatory cytokines being occurred, the host becomes immunosuppressed, which can lead to death [13, 14]. A recent large observational study of sepsis revealed mortality highest when proinflammatory and anti-inflammatory cytokine levels are high [15]. These findings demonstrate that limiting both proinflammatory and anti-inflammatory cytokines should be considered in treating sepsis.

*Bai-Hu-Tang* (BHT, also known as *White Tiger Decoction*), a widely used Chinese medicine formula composed of *Shi-Gao* (*Gypsum Fibrosum*), *Zhi-Mu* (*Rhizoma Anemarrhenae*), *Zhi-Gan-Cao* (Radix Glycyrrhizae Preparata), and *Geng-Mi* (nonglutinous rice), has been extensively used in the early stage of acute infection with systemic inflammation for two thousand years in Chinese medicine [16–18], yet no empirical studies conducted. We explored treatment effects of BHT against cecal ligation and puncture- (CLP-) induced sepsis in rats. Since plasma levels of interleukin-6 (IL-6) and interleukin-10 (IL-10) were demonstrated as good parameters to predict the outcome of sepsis in our previous study [19], survival rate and plasma levels of IL-6 and IL-10 of rats were measured in the present study. Furthermore, mean arterial pressure (MAP) and heart rate (HR) were also measured to monitor the development and progression of sepsis.

## 2. Materials and Methods

### 2.1. Drugs and Preparation

*Bai-Hu-Tang* concentrated extract powder is composed of four ingredients: *Shi-Gao* (*Gypsum Fibrosum*), *Zhi-Mu* (*Rhizoma Anemarrhenae*), *Zhi-Gan-Cao* (Radix Glycyrrhizae Preparata), and *Geng-Mi* (nonglutinous rice) with the proportion of 3 : 8 : 1 : 4. We purchased the formula from a renowned GMP manufacturer of concentrated herbal extracts which conforms to international standards (*Sun-Ten* Pharmaceutical CO., Taipei; its products can be purchased in Asia, Europe, and North America.). When in use, BHT was prepared by mixing 1 gram of extract powder with 5 mL of warm sterile distilled water (30°C).

### 2.2. Animal Model

A total of 36 male Sprague-Dawley (SD) rats, weighing 250 to 300 g, were obtained from BioLASCO Taiwan Co. Ltd. and then maintained at a constant 25°C with free access to pelleted food and water in a room with 12 h light/dark cycle. Sepsis was induced by CLP as previously described [19, 20]. Briefly, rats were anesthetized with ether, with blood samples obtained from the tail artery. After that, a 2 cm midline incision was made, and then the cecum was exteriorized. Ligation was performed below the ileocecal valve without causing intestinal obstruction. Ligated cecum was punctured twice with a 20-gauge needle at the antimesenteric surface and then gently squeezed to extrude adequate amount of stool through two puncture wounds. The bowel was then replaced in the peritoneal cavity and abdomen closed in layers with 3-O silk sutures. Finally, warm saline (3 mL/100 g body weight, 37°C) was subcutaneously injected [20]. Sham-operated rats underwent the same treatment, except for ligation and puncture procedures.

The right carotid artery was then cannulated by PE-50 tubing (Clay Adams, Parsippany, NJ, USA) equipped with a heparin lock filled with heparin saline to permit blood pressure measurement, drawing of blood samples, and infusion of fluids. This catheter was passed through the subcutaneous tunnel to the back of the neck. Finally, rats were placed in cages with warm light and allowed to recover for half an hour; serial MAP and HR were measured and recorded by MP100 System (Biopac Inc., Santa Barbara, CA, USA) at 1, 4, 8, and 12 hrs postoperatively by inserting the probe into the catheter via the heparin lock. Experimental protocols were approved by the Animal Experiment Committee of China Medical University and conducted according to the American Physiological Society guiding principles for the care and use of laboratory animals.

### 2.3. Study Groups

Thirty-six rats were randomly divided into six experimental groups of six rats each. According to prior study, after being subjected to CLP, the hyperdynamic state developed within 10 hrs; rats became obviously ill looking 12 hrs postoperatively [20]. Timing of early oral administration of BHT was thus set at 6 hrs postoperatively within the progress of the hyperdynamic state in our study because BHT is applied clinically when the following symptoms developed in the early stage of infectious diseases: high fever, presence of sweating, thirst, and flooding big pulse [17]. The time points we defined as early and late interventions at 6 hrs and 12 hrs postoperatively, respectively, were consistent with current international opinions [21, 22]. This design was also aimed to compare the effects on survival between “early” and “late” administration of BHT.  Table 1 details the intervention established in each group.

### 2.4. Survival Studies

All rats were monitored for three days postoperatively, those surviving for more than three days categorized in the survival group and the remainder in the nonsurvival group.

### 2.5. Collection of Serum Samples and Cytokine Assay

Before CLP and catheterization, a 0.5 mL blood sample was drawn from the tail artery to establish baseline value measurement of cytokines. After CLP and catheterization, 0.5 mL blood samples were taken from the heparin lock of the carotid artery catheter 4, 8, and 12 hrs later. Normal saline (0.5 mL, 37°C) was injected into the artery to replace blood volume each time. The blood samples were placed in 1.5 mL microtubes and centrifuged at 9000 rpm for 5 min at 25°C to separate plasma. Serum samples were then removed and stored at −80°C for further cytokine assay. Serum levels of IL-6 and IL-10 were measured by the enzyme-linked immunosorbent assay (ELISA) via a commercially available kit (R&D systems, MN55413, Minneapolis, MN, USA) according to manufacturer's specifications.

### 2.6. Statistical Analysis

Log-rank test with Bonferroni adjustment for multiple comparisons analyzed differences in survival among all groups.

Comparisons of MAP, HR, and plasma levels of IL-6 and IL-10 were analyzed only among the sham-control, sepsis-control, CLP + BHT-L_6_, and CLP + BHT-H_6_ groups since we did not measure these four observation parameters in the CLP + BHT-L_12_ and CLP + BHT-H_12_ groups. Because MAP, HR, and plasma levels of IL-6 and IL-10 were not normally distributed, Kruskal-Wallis was used for overall comparison and then Mann-Whitney *U* with Bonferroni adjustment for multiple comparisons was adopted to detect the differences of these parameters at baseline, and at 4, 8, and 12 hrs postoperatively among groups.

To examine the dose effect of BHT on survival and cytokine changes, we first derived an ordinal variable by coding the sepsis-control group as “0,” early administration of low-dose BHT (CLP + BHT-L_6_) as “0.5,” and early administration of high-dose BHT (CLP + BHT-H_6_) as “1.” Second, we defined changes of IL-6 and IL-10 by subtracting plasma levels of cytokines at baseline from those at 12 hrs postoperatively. All cytokine change values were natural log transformed. Then we applied simple linear regression to correlate doses of BHT with changes of IL-6 and IL-10. Finally, we adopted Cox proportional hazards models to explore how doses of BHT correlated with survival through modulating cytokines. Statistical analyses used SPSS version 14.0 for Windows (SPSS, Chicago, IL, USA); *P* value < 0.05 was considered statistically significant.

## 3. Results

Totally, 36 rats were observed. However, two were excluded due to catheter obstruction. Therefore, the study population comprised 34 rats.

### 3.1. Effects of BHT on Survival

**Figure 1:**
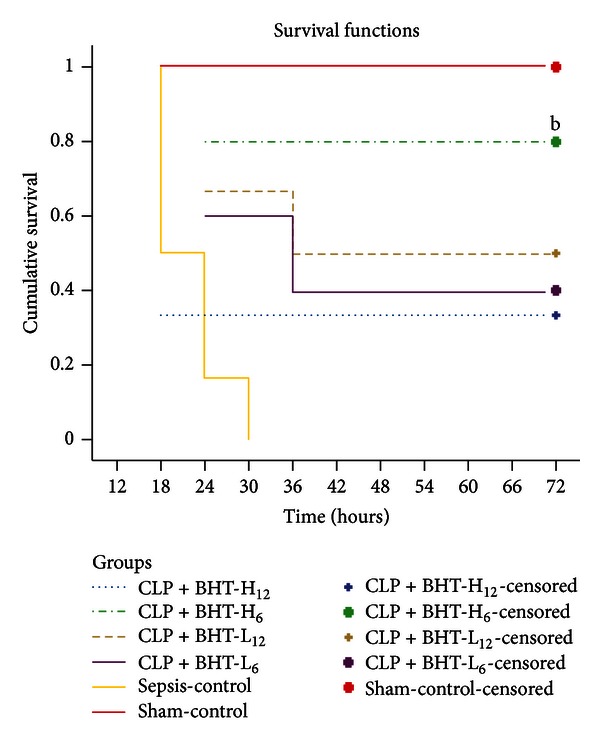
Kaplan-Meier survival curves of all groups. Sham-control group received sham operation and distilled water; the remaining groups received cecum ligation and puncture (CLP). All CLP rats received *Bai-Hu-Tang* (BHT), except for those in the sepsis-control group, which received distilled water. CLP + BHT-L_6_: low dose of BHT (0.45 g/kg) administered 6 hrs postoperatively. CLP + BHT-H_6_: high dose of BHT (0.9 g/kg) administered 6 hrs postoperatively. CLP + BHT-L_12_: low dose BHT (0.45 g/kg) administered 12 hrs postoperatively. CLP + BHT-H_12_: high-dose of BHT (0.9 g/kg) administered 12 hrs postoperatively. All rats were monitored for three days postoperatively for survival study. Notice that the survival was highest in the CLP + BHT-H_6_ group. ^b^ *P* < 0.05, compared to sepsis-control group.

No rats died until 18 hrs postoperatively. Overall test for comparing survival functions of each group was significant (*P* < 0.05). Kaplan-Meier survival curves are shown in Figure 1. The sham-control group had a 100% survival rate, the sepsis-control group 0% in survival. Survival rates were 40% for CLP + BHT-L_6_, 80% for CLP + BHT-H_6_, 50% for CLP + BHT-L_12_, and 33% for CLP + BHT-H_12_. Survival rate was significantly higher in the CLP + BHT-H_6_ group (80%) than in the sepsis-control group (*P* < 0.05).

### 3.2. Effects of BHT on Plasma Levels of IL-6 and IL-10

**Figure 2:**
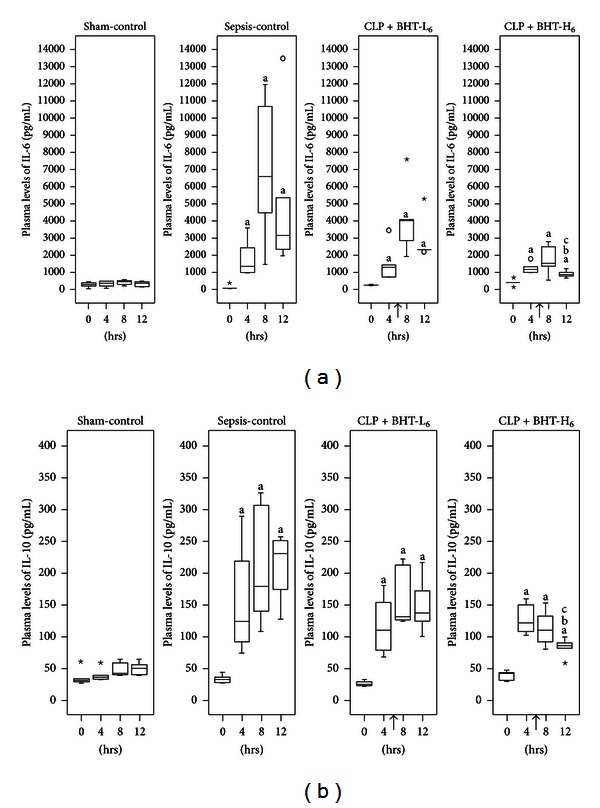
Effects of *Bai-Hu-Tang* on plasma levels of IL-6 (a) and IL-10 (b). Plasma levels of IL-6 and IL-10 in the sham-control, sepsis-control, CLP + BHT-L_6_, and CLP + BHT-H_6_ groups at 0, 4, 8, and 12 hours postoperatively were shown above. Boxes denote 25th and 75th percentile; horizontal lines in boxes indicate the median. Outliers are displayed as circles (○) and extreme values as stars (⋆). “↑”: BHT was administered at 6 hrs postoperatively. Notice that the elevations of IL-6 and IL-10 levels were both reduced after the administration of BHT and it was most prominent in the CLP + BHT-H_6_ group. ^a^ *P* < 0.05, compared with sham-control group. ^b^ *P* < 0.05, compared with sepsis-control group. ^c^ *P* < 0.05, compared with CLP + BHT-L_6_ group.

Figure 2 illustrates changes in plasma levels of IL-6 and IL-10, that is, similar to all groups at baseline and both increasing significantly after CLP in the sepsis-control, CLP + BHT-L_6_, and CLP + BHT-H_6_ groups as compared with sham-control group (^a^ *P* < 0.05).

Early administration of high-dose BHT (CLP + BHT-H_6_) significantly reduced elevations of IL-6 and IL-10 as compared with the sepsis-control group at 12 hrs postoperatively (^b^ *P* < 0.05, IL-6: 868.24 versus 3177.64 pg/mL; IL-10: 85.35 versus 229.45 pg/mL). Likewise, compared with low-dose BHT administration group, rats in the high-dose group had significantly lower plasma levels of IL-6 and IL-10 at 12 hrs postoperatively (^c^ *P* < 0.05, IL-6 median: 868.24 versus 2346.94 pg/mL, IL-10 median: 85.35 versus 136.32 pg/mL).

### 3.3. Effect of BHT on MAP and HR

**Figure 3:**
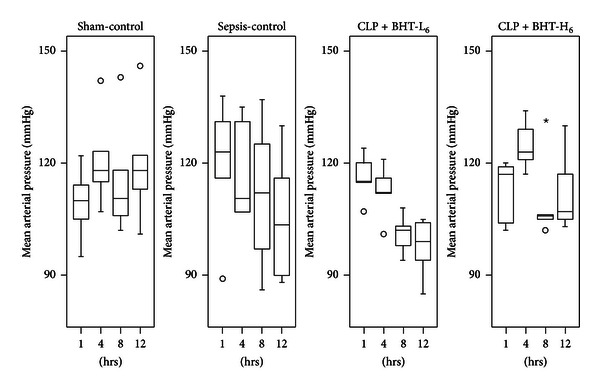
Effects of *Bai-Hu-Tang* on mean arterial pressure (MAP). MAP in sham-control, sepsis-control, CLP + BHT-L_6_, and CLP + BHT-H_6_ groups at 0, 4, 8, and 12 hours after operation were expressed as the box plots. Boxes denote the 25th and 75th percentile; horizontal lines in the box indicate the median. Outliers are displayed as circles (○) and extreme values as stars (⋆). No significant difference was noted at any time point among the four groups.

**Figure 4:**
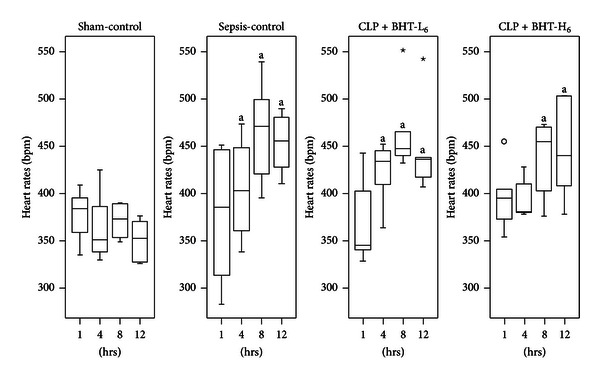
Effects of *Bai-Hu-Tang* on heart rates (HR). HR in the sham-control, sepsis-control, CLP + BHT-L_6_, and CLP + BHT-H_6_ groups at 0, 4, 8, and 12 hours after operation were shown above. Boxes denote 25th and 75th percentile; horizontal lines in the box indicate the median. Outliers are displayed as circles (○) and extreme values as stars (⋆). Notice that HR elevated in all groups after CLP. ^a^ *P* < 0.05, compared with sham-control group.

No significant difference in MAP arose among four groups at baseline and postoperatively (Figure 3). HR were similar in all groups at baseline and increased significantly after CLP, as compared with the sham-control group (^a^ *P* < 0.05). No significant difference appeared for any BHT treatment group versus sepsis-control group (Figure 4).

### 3.4. Association among BHT, Cytokine Changes, and Survival Rate

Table 2 plots linear regression model for association between different doses of BHT and cytokine changes. We found significant negative correlation between a high dose of BHT and change in IL-6 and IL-10 (*β* = −2.13 and −1.41, resp., both *P* < 0.001). The model thus proved effects of BHT on changes of IL-6 and IL-10 as inverse and dose-dependent (*β* = −2.09, −1.39, *P* *for trend *<0.001).  Table 3 displays results of Cox regression models. Before adjustment, high dose BHT manifested negative correlation with risk of death (hazard ratio: 0.08; 95% confidence interval (CI): 0.01–0.73) (*P* < 0.05); changes in IL-6 and IL-10 both exhibited significant positive correlation with individual risk of death (hazard ratio: 3.58 and 4.56; 95% (CI): 1.50–8.56 and 1.50–13.84) (*P* < 0.05). Accounting for diverse BHT doses and cytokine changes, IL-6 variation was the sole predictor for survival, estimated at nearly fourfold greater risk of death (adjusted hazard ratio: 3.87; 95% (CI): 1.03–14.53) (*P* < 0.05).

## 4. Discussion

To our knowledge, this is the first study to explore treatment effects of BHT on survival and cytokine regulation in CLP-induced septic rats. Our results show early administration of high-dose BHT significantly improving survival by reducing IL-6 level. Although IL-10 was simultaneously reduced, modulating effects of BHT on IL-10 did not have significant effects on survival. In addition, we revealed effects of BHT on cytokine changes as dose dependent.

The study design of this study is based on our previous report [19], in which the prognosis of septic rats was followed up for 3 days after CLP, and IL-6 and IL-10 were found to be good predictors of sepsis mortality. Therefore, this time we adopted the same experiment protocol and administered BHT only one time after sepsis induction, in order to clearly evaluate the effects of BHT on both cytokines changes and survival rate. The protocol to monitor the septic rats for the first 3 days after sepsis induction is the same as the method reported in similar studies of sepsis [23, 24]. Besides, once the septic rats can survive more than 3 days, most of them will survive thereafter.

There has been no satisfying improvement in finding new therapies to decrease mortality of sepsis in the past 20 years. Many clinical trials and experiments suffered from design flows [25], for example, key biological information not accessed before initiating trial of an experimental agent [25]. Target biomarker should be present at the time of study entry. Serial measurement should better evaluate the immune status of sepsis [25]. Hence, our study measured baseline levels of cytokines before operation and obtained time serial plasma cytokines levels later, thus confirming IL-6 and IL-10 plasma levels as elevated already before BHT administration; their change patterns followed treatment intervention. These data lent insight into dynamic BHT immune-modulating effects. Simultaneously, time serial MAP and HR were measured, enabling us to gauge development and progression of sepsis in CLP rats.

Recently, both IL-6 and IL-10 elevations were found to be significantly correlated with mortality of sepsis [15, 26]. Peng et al. revealed hemoadsorption decreasing risk of death from sepsis by removal of both IL-6 and IL-10 [27]. Our results concurred with these findings, yet there were several key features in our study. First, oral BHT administration was not as invasive as hemoadsorption or hemofiltration. Second, unlike hemoadsorption or hemofiltration applied in treating late-stage sepsis, appropriate treatment timing of BHT is at early stages of sepsis. Third, while proinflammatory cytokines, including IL-1, tumor necrosis factor alpha (TNF-*α*), and IL-6, have been shown to induce cardiac myocyte apoptosis and necrosis and may cause damage to other organs, expression of inflammatory cytokines during specific response is not entirely deleterious. Inflammatory cytokines are necessary for limiting and eliminating local infections and increasing host survival [28, 29], for example, IL-6 required for successful outcome in sepsis, given its ability to promote hepatocyte recovery and regeneration [30]. These findings point to appropriate downregulation, albeit not extreme suppression, of proinflammatory cytokines as beneficial in treating sepsis. Our study results showed that the BHT administration decreases elevation of IL-6 after CLP without totally blocking the expression of IL-6, which might not make the host suffer from immunosuppression.

On the other hand, magnitude of IL-10 response appears to correlate with both severity of inflammatory insult and plasma concentration of proinflammatory cytokines, such as TNF-*α* [31]. We found that IL-10 simultaneously reduced following early administration of high-dose BHT, but with no direct cause and effect between BHT administration and level of IL-10. These findings imply reduction of proinflammatory cytokine in early-stage sepsis as sequentially downregulating anti-inflammatory cytokine, IL-10. Hence, the mechanism by which BHT modulated IL-6 and whether BHT can modulate TNF-*α* or other proinflammatory cytokines should be evaluated in the future.

This study had several limitations. First, due to limited amount of blood volume we could draw from rats each time, we only observed plasma level of proinflammatory cytokine IL-6 and anti-inflammatory cytokine IL-10, with the main focus chosen from the literature review and based on results of our prior study [15, 19, 26, 32]. Second, to probe treatment effects of BHT, our intervention only included BHT administration with limited fluid supplement (Normal Saline, 3 mL/100 g body weight). Thus, we could not evaluate treatment effects of BHT applied in combination with current clinical therapies like antibiotics, fluid resuscitation, or inotropics. Third, before formally performing the experiment described in this manuscript, pilot studies were conducted to confirm the optimal dose of BHT adopted. The results of these pilot studies revealed the same trend that the group of septic rats which received BHT had higher survival rate in a dose-dependent manner. Due to the limitation of grant resources, the sample size of each group in this experiment only received the average number of animal experiments [23, 24]. The relative small sample size is a limitation of our study, but the dose-dependent effects of BHT on both the survival rate and circulating IL-6 and IL-10 still provide evidence of BHT as a new treatment option for sepsis.

## 5. Conclusions

For centuries, based on theories of traditional Chinese Medicine, BHT has been considered effective in treating early stage severe infection with systemic inflammation. Results of our study afforded scientific evidence for BHT in treating sepsis by a CLP model and its effects on cytokine modulation: early administration of high-dose BHT markedly improved survival by directly reducing plasma level of IL-6, and simultaneously decreased IL-10 was observed. Our results suggest BHT as a novel complementary therapeutic candidate in treating early sepsis besides antibiotic, fluid resuscitation, and inotropic therapy, though combined effects of BHT with these treatments still need further evaluation.

## Figures and Tables

**Table 1:** Study groups and their interventions.

Groups	Model	Administration (timing)
Sham-control (*n* = 6)	Sham operation	Distilled water 1 mL as vehicle (6 hrs after operation)
Sepsis-control (*n* = 6)	CLP	Distilled water 1 mL as vehicle (6 hrs after CLP)
CLP + BHT-L_6_ (0.45 g/kg) (*n* = 6)	CLP	BHT 0.5 mL + distilled 0.5 mL (6 hrs after CLP)
CLP + BHT-H_6_ (0.9 g/kg) (*n* = 6)	CLP	BHT 1 mL (6 hrs after CLP)
CLP + BHT-L_12_ (0.45 g/kg) (*n* = 6)	CLP	BHT 0.5 mL + distilled 0.5 mL (12 hrs after CLP)
CLP + BHT-H_12_ (0.9 g/kg) (*n* = 6)	CLP	BHT 1 mL (12 hrs after CLP)

CLP: rats received cecal ligation and puncture.

BHT-L_6_: low dose of BHT was orally administered 6 hrs postoperatively.

BHT-H_6_: high dose of BHT was orally administered 6 hrs postoperatively.

BHT-L_12_: low dose of BHT was orally administered 12 hrs postoperatively.

BHT-H_12_: high dose of BHT was orally administered 12 hrs postoperatively.

**Table 2:** Linear regression model of association between different doses of BHT and cytokine changes.

	Change of IL-6^b^			Change of IL-10^b^		
	*β*	*P* value	*P* for trend	*β*	*P* value	*P* for trend
Dose of BHT	−2.09		<0.001*	−1.39		<0.001*
High dose (1)^a^	−2.13	<0.001*		−1.41	<0.001*	
Low dose (0.5)^a^	−0.39	0.295		−0.38	0.081	

^a^Ordinal variable: coding the early administration of high-dose BHT (CLP + BHT-H_6_) as “1,” and the early administration of low-dose BHT administration (CLP + BHT-L_6_) as “0.5”.

^b^changes of IL-6 and IL-10: subtracting the plasma levels of cytokines at baseline from those at 12 hrs postoperatively.

All cytokine changed values were natural log transformed.

*P < 0.05.

**Table 3:** Cox proportional hazards models of survival rates for different doses of BHT and changes of IL-6 and IL-10.

Covariate	Unadjusted		Adjusted^+^	
	HR (95% CI)	*P* value	HR (95% CI)	*P* value
Dose of BHT				
High dose (1)^a^	0.08 (0.01–0.73)	0.025*	0.56 (0.02–20.17)	0.751
Low dose (0.5)^a^	0.26 (0.06–1.19)	0.083	0.25 (0.05–1.40)	0.115
Sepsis control (0)^a^			Reference	

Cytokine changes				
Change of IL-6^b^	3.58 (1.50–8.56)	0.004*	3.87 (1.03–14.53)	0.045*
Change of IL-10^b^	4.56 (1.50–13.84)	0.007*	0.82 (0.10–6.91)	0.851

^a^Ordinal variable: coding the early administration of high-dose BHT (CLP + BHT-H_6_) as “1,” and the early administration of low-dose BHT administration (CLP + BHT-L_6_) as “0.5”.

^b^Changes of IL-6 and IL-10: subtracting the plasma levels of cytokines at baseline from those at 12 hrs postoperatively.

All values were natural log transformed.

Unadjusted: univariate Cox proportional hazards model.

Adjusted^+^: for different doses of BHT, changes of IL-6 and IL-10.

HR: hazard ratio, CI: confidence interval, *P< 0.05.
